# Ferroptosis-Related Genes in Neurodevelopment and Central Nervous System

**DOI:** 10.3390/biology10010035

**Published:** 2021-01-06

**Authors:** Soo-Whee Kim, Yujin Kim, Sung Eun Kim, Joon-Yong An

**Affiliations:** 1 Department of Biosystems and Biomedical Sciences, College of Health Sciences, Korea University, Seoul 02841, Korea; rlatngnl@korea.ac.kr (S.-W.K.); kyujin9805@korea.ac.kr (Y.K.); 2 Department of Integrated Biomedical and Life Sciences, College of Health Sciences, Korea University, Seoul 02841, Korea

**Keywords:** ferroptosis, lipid peroxides, iron, brain development, neurological disorder, CNS

## Abstract

**Simple Summary:**

Ferroptosis is a recently defined form of regulatory cell death. To get a grasp of the prospective role of ferroptosis in the brain development, we collated ferroptosis-related genes and investigated their involvement underlying the ferroptosis mechanism and the association with neurological disorders. With the application of ferroptosis as a therapeutic intervention target for such diseases garnering attention, we assume that understanding the genetic architecture of ferroptosis is indispensable.

**Abstract:**

Ferroptosis, first introduced as a new form of regulated cell death induced by erastin, is accompanied by the accumulation of iron and lipid peroxides, thus it can be inhibited either by iron chelators or by lipophilic antioxidants. In the past decade, multiple studies have introduced the potential importance of ferroptosis in many human diseases, including cancer and neurodegenerative diseases. In this review, we will discuss the genetic association of ferroptosis with neurological disorders and development of the central nervous system.

## 1. Introduction

Cell death is an essential process for normal development and tissue homeostasis in countless organisms, including humans. Apoptosis, the most well-studied form of programmed cell death, is associated with cellular changes such as shrinkage, blebbing, chromatin condensation, and caspase activation, whereas necrosis was broadly thought to be a type of accidental cell death that results from cell rupture. However, it is now settled that many cell death processes that morphologically resemble necrosis are regulated by certain modules and pathways. Among these, ferroptosis is a newly defined form of iron-dependent cell death that is executed by the accumulation of lipid peroxides. It is distinct from apoptosis and other forms of necrosis such as necroptosis or pyroptosis in that it can be blocked by iron chelation or eliminating lipid peroxides. The potential of ferroptosis for therapeutic application in various diseases, especially cancer, has been highly appreciated and is being actively pursued [1]. On the other hand, scientists have recently started to shed light on the implication of ferroptosis in neurodegenerative diseases and brain injury. In fact, ferroptosis shares many features with oxytosis, a death process that occurs in neurons in response to glutamate toxicity [2]. Its association with neurological diseases such as Alzheimer’s disease (AD) [3,4], Parkinson’s disease (PD) [5,6], Huntington’s disease (HD) [7,8], traumatic brain injury (TBI) [9], stroke [10,11], and seizure-induced brain damage [12] has been illuminated. However, the association of ferroptosis in the development of the brain and central nervous system (CNS) has not been examined. Here, we review the genetic background of ferroptosis in the brain development by analyzing ferroptosis-regulating genes in multiple cell types and stages of brain development. Future studies are warranted to see whether ferroptosis has a significant role in the development of the brain and central nervous system, and its explicit mechanism remains to be established.

After Dixon first proposed the concept of ferroptosis in 2012 [13], many researchers have identified multiple genes implicated in the regulation of ferroptosis. In this review, we selected 42 genes involved in the regulation of ferroptosis (FR) from currently published data, and discussed their putative roles in brain development. Following is the list of them; [inducing] arachidonate 12/15/5-lipoxygenase (*ALOX12*, *ALOX15*, *ALOX5*), lysophosphatidylcholine acyltransferase 3 (*LPCAT3*), acyl-CoA synthetase long chain family member 4 (*ACSL4*), spermidine N1-acetyltransferase 1 (*SAT1*), transferrin (*TF*), transferrin receptor (*TFRC*), nuclear receptor coactivator 4 (*NCOA4*), autophagy-related 5/7 (*ATG5, ATG7*), microtubule-associated protein 1 light chain 3 alpha (*MAP1LC3A*), microtubule-associated protein 1 light chain 3 beta (*MAP1LC3B*), dipeptidyl-peptidase 4 (*DPP4*), cytochrome p450 oxidoreductase (*POR*), heme oxygenase-1 (*HMOX1*), voltage-dependent anion channel 2/3 (*VDAC2, VDAC3*), Yes 1-associated transcriptional regulator (*YAP1*), WW domain-containing transcription regulator 1 (*WWTR1*), activating transcription factor 3/4 (*ATF3, ATF4*), and endothelial PAS domain protein 1 (*EPAS1*), [suppressing] solute carrier family 3 member 2 (*SLC3A2*), solute carrier family 7 member 11 (*SLC7A11*), tumor protein p53 (*TP53*), glutamate-cysteine ligase catalytic subunit (*GCLC*), glutathione synthetase (*GSS*), glutathione peroxidase 4 (*GPX4*), solute carrier family 40 member 1 (*SLC40A1*), ceruloplasmin (*CP*), solute carrier family 11 member 2 (*SLC11A2*), ferritin heavy chain 1 (*FTH1*), ferritin light chain (*FTL*), ferritin mitochondrial (*FTMT*), apoptosis-inducing factor mitochondria-associated 2 (*AIFM2*), NEDD4 E3 ubiquitin protein ligase (*NEDD4*), nuclear factor erythroid 2 like 2 (*NFE2L2*), prominin 2 (*PROM2*), hypoxia-inducible factor 1 subunit alpha (*HIF1A*), transcription factor AP-2 gamma (*TFAP2C*), and SP1 transcription factor (*SP1*) [10,14,15,16,17,18,19,20,21,22,23].

## 2. Mechanism of Ferroptosis

**Figure 1:**
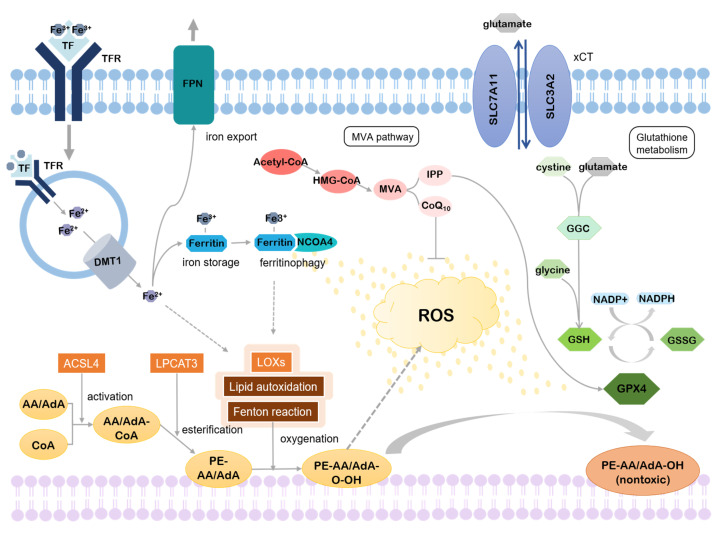
Mechanism of ferroptosis. Iron-dependent accumulation of lipid peroxides promotes ROS production, causing ferroptosis. By-products of the mevalonate pathway and glutathione metabolism, however, can rescue ferroptosis.

Various metabolic pathways are involved in the regulation of ferroptosis. Studies have found that the accumulation of lipid peroxides is essential for the progression of ferroptosis [24]. Intracellular iron concentration is also important. It appears when there is excess iron in cells, reactive oxygen species (ROS) levels, which eventually leads to the initiation of ferroptosis, which also increases [25]. Thus, the regulatory metabolic pathways of ferroptosis can be roughly divided into three: lipid metabolism, iron metabolism, and others (Figure 1).

### 2.1. Lipid Metabolism

#### 2.1.1. Accumulation of Lipid Peroxides

M Lipids, the main components of the intracellular membrane, play important roles in cellular metabolism and signaling. In addition, esterification of lipids into membrane phospholipids (PLs) enables much more complex physiological functions, and oxygenation of PLs facilitates signaling pathways [26]. This, however, results in the accumulation of lipid peroxides and if it exceeds the threshold, this may induce ferroptotic cell death [24]. Thus, lipid peroxidation is a double-edged sword, which can be both beneficial and lethal to the cell depending on the context.

The final product of ferroptotic lipid metabolism, lipid ROS, is the main contributor to the execution of ferroptosis. An increased lipid ROS level may lead to damage in the cellular membrane, ultimately inducing ferroptosis. Although further elucidation is required to understand how ROS induce ferroptosis, three major pathways of ROS production are known: peroxidation of lipid [24,27,28], the Fenton reaction, and autoxidation of lipid [29], all of which require iron. Lei et al. expected that lipid peroxidation is involved in the initiation of ferroptosis, while the Fenton reaction and lipid autoxidation mediates the final step of it [15].

PUFAs, polyunsaturated fatty acids, are highly susceptible to lipid peroxidation [30]. They are esterified into membrane phospholipids by the act of acyl-coA synthetase long chain family member 4 (ACSL4) and lysophosphatidylcholine acyltransferase 3 (LPCAT3) [24,31,32]. Phosphatidylethanolamines (PEs) containing arachidonic acid (AA) or adrenic acid (AdA) are key products of esterification that contribute to ferroptosis [24,31]. After ACSL4 activates AA/AdA to make coenzyme-A-derivatives, AA/AdA-CoA, LPCAT3 esterifies it into membrane PLs, PE-AA/AdA. The ACSL family includes five isoforms of ACSLs, ACSL1, ACSL3, ACSL4, ACSL5, and ACSL6, all of which convert fatty acids into acyl-CoAs [29]. Among them, ACSL4 scored highest in the association with ferroptosis. It has been shown that the double knock-out of GPX4 and ACSL4 can rescue ferroptosis through multiple experiments. This result points out that ACSL4-controlled formation of AA/AdA-CoA is critical for the ferroptosis process. Other ACSLs also have a moderate effect of PUFA activation but only when there is a high concentration of PUFAs.

Esterified PUFAs are further oxidized to phospholipid hydroperoxides, PE-AA/AdA-OOH, either by the Fenton reaction, which is widely observed in nature, or lipid autoxidation, which proceeds by a free radical chain mechanism [24,31]. The Fenton reaction oxidizes ferrous iron with hydrogen peroxide and then reduces back to ferrous iron with another hydrogen peroxide, generating two different ROS. Lipid autoxidation produces a fatty acid radical, which reacts with oxygen to make a peroxyl-fatty acid radical, creating a fatty acid radical again [33]. This cycle continues until two radicals react with each other [34]. Unlike the Fenton reaction and lipid autoxidation, which are both non-enzymatic processes, lipid peroxidation is mediated by several enzymatic effectors. Of note, it was found recently that lipoxygenases (LOXs), which catalyze the dioxygenation of PUFAs, are one of them [24,35]. Many results on LOX inhibitors proved that they can inhibit ferroptosis by disrupting lipid peroxidation, implying the importance of LOXs in ferroptosis promotion. However, not all LOX inhibitors can prevent ferroptosis [28] and this suggests that they might be required just for the initiation stage of ferroptosis.

#### 2.1.2. Inhibition of Lipid Peroxides Reduction

As a high concentration of lipid peroxides can be fatal, cells have evolved to balance out oxidative stress. One of the cell protection strategies against oxidative stress is to reduce lipid peroxides to nontoxic alcohols. Glutathione peroxidase 4 (GPX4) plays a crucial role in this reduction with the help of cofactor glutathione, GSH [1,36]. Thus, inhibition of the activity of GPX4 or GSH biosynthesis makes cells susceptible to ferroptosis. This can be done by directly inactivating GPX4 and GSH or by indirectly regulating their synthesis.

Cysteine, the key ingredient in the synthesis of GSH [14,27], can be imported via cystine-glutamate transporter, system Xc- or synthesized through the transsulfuration pathway [37,38]. L-glutamate and cysteine, combined together by glutamate-cysteine ligase (GCL), form glutamyl-cysteine. Subsequent binding with glycine by glutathione synthase (GSS) produces GSH. Nicotinamide adenine dinucleotide phosphate (NADPH) is required to regenerate GSH from glutathione disulfide (GSSG) [39]. On the other hand, isopentenyl diphosphate (IPP), byproduct of the mevalonate (MVA) pathway with coenzyme Q10 (CoQ10), and selenium are required for the synthesis of GPX4 [40,41].

The intracellular concentration of cystine is mainly controlled by system Xc-. This antiporter is a heterodimer composed of light chain SLC7A11 and heavy chain SLC3A2, which are linked by disulfide. Cystine is imported by system Xc- in exchange for glutamate in a 1:1 ratio [37]. Therefore, if there is a high concentration of glutamate in the extracellular part, cystine import is suppressed, inhibiting GSH synthesis [13]. Ferroptosis inducers such as erastin, sorafenib, and sulfasalazine act by targeting system Xc-, which leads to GSH depletion and ferroptosis. Some cells, however, are able to synthesize cysteine from methionine through the transsulfuration pathway. In this pathway, after homocysteine is synthesized from methionine, it is interconverted to cysteine under the act of intermediate cystathionine, including cystathionine beta synthase (CBS) and cystathionine gamma lyase (CGL). Production of cysteine via transsulfuration pathway is enough to maintain adequate concentrations in the cytoplasm, thus conferring resistance to ferroptosis induced by system Xc- inhibitors [42], described above.

The MVA pathway produces IPP, which is required for the maturation of selenocysteine tRNA [40]. As selenocysteine tRNA plays a pivotal role in GPX4 synthesis [43], IPP production of the MVA pathway inhibits ferroptosis. Another product of the MVA pathway is CoQ10. β-Hydroxyl β-methylglutaryl-CoA (HMG-CoA) formed from acetyl-CoA by HMG-CoA synthase is converted into mevalonate, which is then synthesized as CoQ10 with HMG-CoA reductase. CoQ10, a powerful antioxidant in membranes, subsequently represses ferroptosis under the oxidative stress [44].

### 2.2. Iron Metabolism

Accessibility of iron is vital for the induction of ferroptosis [30]. Even the term “ferroptosis” is named after its iron dependence [13]. Extracellular iron is imported into cells by transferrin (TF) and transferrin receptor (TFRC) by endocytosis. After reduction to ferrous iron (Fe^2+^) in the endosome, iron is released into the cytoplasm through divalent metal transporter 1 (DMT1) and excess iron is stored by ferritin. Binding with NCOA4, ferritin can be degraded, which leads to the release of stored iron. If ferritins are saturated, then excess iron is exported through ferroportin (FPN).

It is still controversial whether excess ROS produced by iron contributes to tumorigenesis [45,46], which is the counteraction of ferroptosis because ferroptotic cell death inhibits the proliferation of tumor cells. Apart from iron’s involvement in tumor pathology, it is evident that a higher concentration of intracellular ferrous iron (Fe^2+^), harmful for its ease of electron transfer, is followed by ferroptosis. This explains the reason why ferroptosis can be prevented by iron chelators [13] and TF, TFRC, and NCOA4 positively regulate ferroptosis [47,48] while ferritin negatively regulates ferroptosis [23,49,50,51].

For systemic level, intracellular iron is regulated with or without hepcidin, which is secreted from the liver. Depending on the concentration of iron, binding affinity between hepcidin and FPN differs and this mediates iron export. On the other hand, for cellular level, IRP1-IRE binding activity controls iron uptake, sequestration and export. Upregulation of IREB2, the main transcription factor of iron metabolism, increases sensitivity toward ferroptosis [13]. Additionally, recent studies have found that heat shock protein family B member 1 (HSPB1) and CDGSH iron sulfur domain 1 (CISD1) are also associated with iron metabolism, affecting ferroptosis sensitivity [44,52].

### 2.3. Others

In addition, there are several other regulatory pathways that regulate ferroptosis. The autophagy receptor p62, for example, activates nuclear factor erythroid 2-related factor 2 (NRF2) by inactivating Kelch-like ECH-associated protein 1 (Keap1) [53,54]. As a result, the downstream genes of *NRF2* related to antioxidant activity, iron metabolism, and glutathione synthesis like NADPH quinone dehydrogenase 1 (*NQO1*), heme oxygenase (*HO1*), and ferritin heavy chain 1 (*FTH1*) are induced, inhibiting ferroptosis [55]. Nevertheless, this mechanism has yet to be solved in that ferroptosis inducers aid in p62′s binding to Keap1 to competitively hinder the interaction between Keap1 and NRF2, facilitating NRF2 in hepatocellular cancer (HCC) [55].

The tumor protein p53, otherwise, is known to induce ferroptosis either by downregulating SLC7A11 [56] of system Xc- or by p53-SAT1-ALOX15 pathway. But when it comes to colorectal cancer (CRC), p53 interferes with dipeptidyl-peptidase-4 (DPP4), which is involved in lipid peroxidation, therefore inhibiting ferroptosis [19]. It is assumable that p53 has varying effects depending on the cell types. Also, ferroptosis suppressor protein 1 (FSP1) inhibits ferroptosis by promoting CoQ10 regeneration in the MVA pathway with NADPH [57,58].

## 3. Association with Neurological Diseases

With extensive findings on aberration of iron homeostasis concomitant of numerous neurological diseases, ferroptosis is gaining increasing attention as a new therapeutic target. For example, ferroptosis has been implicated with Friedreich’s ataxia, a form of autosomal recessive neurodegenerative disease caused by reduced levels of frataxin, a mitochondrial protein involved in iron–sulfur cluster synthesis. This leads to mitochondrial iron accumulation and increased oxidative stress, which can lead to ferroptosis. Patient-derived fibroblasts showed higher sensitivity to ferroptosis-inducing drugs, suggesting that ferroptosis may be a novel therapeutic target for Friedreich’s ataxia [59]. Similarly, as dysregulation of glutathione homeostasis is implicated in the progression of neurodegenerative diseases, such as Alzheimer’s disease (AD), Parkinson’s disease (PD), and Huntington’s disease (HD), ferroptosis may be involved in these processes as well [60].

To determine the association of ferroptosis with neurological diseases, including neurodegenerative diseases and brain injury, we screened the literature for genetic factors under such mechanisms. According to genome-wide association study (GWAS), there are four genes that are related to neurological diseases among the 42 ferroptosis genes we selected. First of all, on *TF* the association with alcohol intake [61,62], sleep disorder in autism spectrum disorder (ASD) patients [63], and depression in adolescents [64] was reported. *EPAS1* is indicative of AD [65,66] and amyotrophic lateral sclerosis (ALS) [67] in a way that is downregulated and upregulated, respectively. In addition, *MAP1LC3B* was found to be implicated in major depressive disorder (MDD) [68] and volume of the lateral ventricles (LVs) [69]. On the other hand, the sole suppressing FR gene *SLC7A11* out of four genes, known to be correlated to alcohol dependence [70], is downregulated in PD [71] and overexpressed in untreated multiple sclerosis (MS) [72]. Although those discoveries are of significance, whether they are experimentally replicable should be examined.

## 4. Brain Development

The maturation of the CNS occurs in six major well-defined stages, with each spanning through the restricted period, yet some of which are protracted over the course of brain development. Followed are six stages of CNS maturation: dorsal induction, ventral induction, proliferation, migration, organization, and myelination.

By the end of gastrulation, three germ layers, endoderm, mesoderm, and ectoderm, have been established. Stem cells in the endoderm, the innermost layer of the embryo, give rise to structures of the digestive and respiratory system, while in the intermediate mesoderm they give rise to structures such as muscle, bone, and connective tissues. On the other hand, stem cells in the outermost layer, the ectoderm, give rise to epidermis and the nervous system. Neurulation occurs within the ectoderm, bending the neural plate to form the neural tube, the origin of the entire CNS. During neurulation, dorsal induction (3–4 post conceptional weeks (pcw)) and ventral induction (5–10 pcw) occur sequentially. In dorsal induction, the neural tube closes and the spinal cord is formed. In the second stage, ventral induction, the neural tube develops into the structures of the face and brain. The hollow center of the early neural tube, also known as ventricular zone (VZ), is where neuroepithelial cells (NECs) are found. To supply neurons and glial cells required for the brain organization, NECs undergo rapid symmetric cell divisions. It has been demonstrated that patterning of the early neocortex arises from NECs with a different combination of transcription factors. Especially, two signaling molecules of the neocortical proliferative zone, empty spiracles homeobox 2 (Emx2) and paired box protein 6 (Pax6), are known to play a key role in neural patterning, restricting the fate of NECs to produce appropriate progenitors [73,74].

Subsequent stages are accompanied by the prominent morphological change of the brain. Starting from smooth structure, the brain acquires the folding pattern with gyri and sulci through the development [75]. After primary sulci emerge between 8–26 pcw, secondary sulci form between 30–35 pcw [76]. Tertiary sulci appear at 36 pcw and their formation continues well after the birth [76]. As the development of tertiary sulci is deeply associated with that of cognition, this period is critical [77,78,79,80,81,82]. In contrast, at cellular level, NECs lose their tight junctions and are transformed into radial glial cells (RGCs). As a result of asymmetric division of RGCs, not only do RGCs proliferate but also various types of neural progenitors are made. From them, neurons and glial cells are differentiated and the differentiation begins in both prenatally. However, while proliferation of neuroblast culminates from 8 to 16 pcw, that of glioblast reaches its peak from 5 to 12 months postnatally. As cells are born, they migrate from the proliferative zone to the final destination in an “inside-out” manner, the next wave of migrating cells replacing the earlier one [83]. Eventually, the cortex is composed of six layers, each containing different types of neurons. Studies have found that there is a transition of mode of neuron migration as development proceeds. In early phase of development, neurons migrate by somal translocation where their cell body extends toward the pial surface and then the nucleus moves up [84,85]. When the brain gets bigger, however, radial glial cells act as a scaffold to guide neuron migration [84,85,86].

The fifth stage of CNS development is the organization of neural networks, which starts at six months of gestation and continues in the postnatal period. This process involves the projection of axons and dendrites from neurons, genesis of synapses, and some regressive events. Once a neuron enters the stage of maturation with extended axons and dendrites, inputs from other neurons are received through dendrites and this information is sent down the axon. Also, there is a structure called a growth cone at the tip of each axon and it elongates toward a hospitable environment. In this way, the axon reaches its target, making synapses through which neural cells communicate with each other. It is well known that early brain development leads to the overproduction of neurons, glial cells, and synapses. Thus, the organization of the CNS inevitably entails some regenerative events including apoptosis and synaptic pruning to optimize neural circuits. The loss of neural cells and synaptic connections caused by them amounts up to over 50%. This occurs mainly prenatally in the neuronal populations and postnatally in the glia populations. Myelination begins at six months of gestation as well and lasts into adulthood. This final phase, the ensheathment of axons with myelin, is performed by oligodendrocytes in the CNS. Since it insulates axons, the speed of transmission is accelerated. It is worth noting that both organization and myelination are prolonged well after the birth, emphasizing the importance of postnatal brain development.

## 5. Ferroptosis in Brain Development

### 5.1. Ferroptosis Genes in Cortical Development

Since high-throughput sequencing technologies have allowed deep gene expression profiling at a genome-wide scale, there has been a range of transcriptomic data sets for developing human brains [87,88,89]. Brain-expressed genes show distinct spatiotemporal patterns across the developmental stages and brain regions, well characterized by a gene co-expression network. Within a gene co-expression network, genes of similar biological processes, regulatory features, cellular composition, or disease association are highly correlated as to their expression profiles, and group into a small network, called a module (M).

The BrainVar data set, the largest transcriptomic data set of 176 human dorsolateral prefrontal cortex (hDLPFC) samples to date, provides 19 modules of the gene co-expression network underlying the developing prefrontal cortex [89]. Of 42 ferroptosis genes, 22 were detected in these modules: [inducing] *VDAC3, POR, ATF4, VDAC2, NCOA4, LPCAT3, TFRC, TF, HMOX1, MAP1LC3A, EPAS1, YAP1*, and *MAP1LC3B;* [suppressing] *AIFM2, PROM2, FTL, GPX4, SLC3A2, GCLC, NFE2L2,* and *SLC7A11* (Table 1). FR genes were mostly found in modules of non-transitional and postnatal specific genes. For non-transitional modules, the majority of FR genes are involved in RNA processing: *NCOA4, ATF4, VDAC2, VDAC3* (inducing genes), and *GSS* (suppressing gene). With *GSS*, for instance, it has been revealed that an alternative splicing variant of the *GSS* gene causes in-frame deletion of 333bp covering exon 4 and 5 [90].

Among ferroptosis-inducing genes, *NCOA4* was detected in M5; *VDAC3, ATF4,* and *VDAC2* in M6; *POR* in M7; *LPCAT3* in M9; *TFRC* and *MAP1LC3B* in M16; *MAP1LC3A* in M17; *HMOX1* and *YAP1* in M18; and *TF* and *EPAS1* in M19. For ferroptosis suppressing genes, on the other hand, *GSS* was identified as a gene in M6; *PROM2* in M9; *AIFM2* in M12; *FTL, GPX4,* and *SLC3A2* in M14; *NFE2L2* and *SLC7A11* in M18; and *GCLC* in M19. Overall, the composition of modules is mainly explained by those of group 5, followed by group 2 to a lesser extent, and groups 3 and 4 evenly contribute to the rest. It is interesting that M18 and M19 together account for the largest portion of module composition as they are strongly enriched in neurons and non-neuronal cells.

### 5.2. Ferroptosis Genes in Neurons and Non-Neuronal Cells

Under the premise that higher postnatal expression denotes the importance of a gene in the brain development process, we put emphasis on FR genes with postnatal trajectory. In fact, the postnatally augmented expression of FR genes, of M18 and M19 more specifically, in neural and non-neuronal cells also support their major role in the brain development. We divided 10 postnatal FR genes according to their enriched cellular types: *MAP1LC3B, TFRC, MAP1LC3A, EPAS1, TF,* and *GCLC* mainly enriched in neuronal cells, and *HMOX1, YAP1, NFE2L2* and *SLC7A11* enriched in non-neuronal cells.

First, postnatal FR genes are mainly enriched in neuronal cells. Of them all, transferrin and transferrin receptor being the primary source of iron delivery to the brain, their crucial roles have been widely acknowledged [91,92]. Not only do they transport iron across the blood–brain barrier (BBB), they also deliver iron to neurons in the brain. Some studies have affirmed that epithelial cells comprising the BBB can export non-Tf bound iron (NTBI) across abluminal plasma membranes as well [93,94,95]. Nonetheless, widespread expression of *TF* and *TFRC* in brain neurons [96,97] and in some of the non-neuronal cells [98,99,100,101,102,103] corroborates the need of those for maintaining iron homeostasis, reflecting the underlying mechanism that is involved in neurological impairments and brain development. Moreover, though astrocytes acquire iron as a form of NTBI by epithelial cells, ceruloplasmin oxidizes iron and FPN exports it to the extracellular space to recharge apo-TF for iron delivery to neurons [104,105,106].

*EPAS1*, also known as *HIF2A,* is one of the hypoxia-inducible factors (HIFs), heterodimeric transcription factors that control oxygen homeostasis by regulating genes related to glycolysis, erythropoiesis, vascular development, and angiogenesis [107]. They are highly sensitive to intracellular oxygen concentration and, in normoxic condition, undergo degradation by prolyl hydroxylases (PHDs) [108]. In the absence of O₂, however, they become stabilized and modulate the expression level of genes associated with neural cell survival and differentiation [109,110,111,112]. Ko et al. found out that under hypoxic condition, HIF2A binds to promoters of surviving orthologues *birc5a* and *birc5b*, which perform neuroprotective functions in zebrafish during embryogenesis [113]. Considering that depletion of *HIF2A* promotes neuron cell apoptosis and even embryonic death [113,114], these observations can yield starting points to investigate its function in the brain development.

Without doubt, GSH is the most important intracellular antioxidant, thus suppressing the promotion of ferroptosis. There are two rate-limiting enzymes for GSH synthesis; one is GSS and the other is GCL. GCLC is the catalytic subunit of the latter one, which is of particular interest as many studies have validated its involvement in neurological disorders [115,116,117]. Indeed, GCLC knock-out (KO) in mice resulted in the disruption of GSH homeostasis, and this caused neurodegeneration [118,119]. Feng et al. argued that neuronal cell death via mitochondrial damage and cytochrome C pathways might be responsible for it [120]. Besides, there is increasing evidence that MAP1LC3A/B is implicated in the progression of neurodegenerative diseases [121,122]. Impaired autophagy in neurons through genetic disruption of *MAP1LC3A/B* along with *ATG5/ATG7* induced neuropathologic features in dementia with Lewy bodies (DLB) patients [123].

Next, postnatal FR genes are enriched in non-neuronal cells. There are two postnatal FR-inducing genes enriched in non-neuronal cells, *HMOX1* and *YAP1*. HMOX1, also known as HO-1, mediates several key functions in metabolic pathways. Its activity includes protein folding as a chaperone, heme degradation, which may also contribute to ferroptosis, and production of promising antioxidants like biliverdin and bilirubin [124]. Though we still know little about which of these mechanisms takes neuroprotective effect, it has been demonstrated that the upregulation of *HMOX1* expression protects neuronal cells against oxidative stress and even alleviates ischemic stroke [125]. Just as HMOX1, YAP1 is in charge of multiple core functions regarding the regulation of cell proliferation and differentiation. In an observational study, it was revealed that activation of YAP1 together with TAZ is essential for Schwann cell development [126,127].

Likewise, *NFE2L2* and *SLC7A11* are glia cell-enriched genes that suppress ferroptosis. *NFE2L2* encodes NRF2 and NRF2 has proved to be activated in neurons and glial cells, but mainly in astrocytes, in patients with Alzheimer’s disease, Parkinson’s disease, amyotrophic lateral sclerosis, Huntington’s disease, and multiple sclerosis [128]. Subsequent experiments in which deletion of NRF2 was induced resulted in the exacerbation of disease phenotypes [129,130,131]. This finding is in line with the hypothesis that NRF2 is required for abrogation of neurodegeneration. As delineated in Section 2, The Mechanism of Ferroptosis, cystine is the main ingredient for GSH synthesis and imported by system Xc-. *SCL7A11*, the gene codes for system Xc-, is highly expressed in non-neuronal support cells, especially astrocytes. It has been shown that SLC7A11 directs GSH astrocyte–neuron coupling, and it alludes to its neuroprotective role [132,133].

### 5.3. Functional Annotation of Ferroptosis Genes in Brain Development

**Figure 2:**
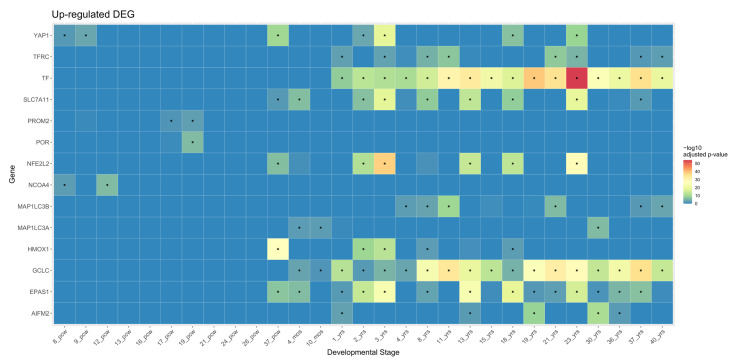
Heatmap of upregulated ferroptosis genes tested against BrainSpan developmental stage. A total of 14 ferroptosis (FR) gene sets displayed significant temporal enrichment in BrainSpan data: *NCOA4, POR, PROM2, AIFM2, TFRC, MAP1LC3B, MAP1LCA, HMOX1, YAP1, NFE2L2, SLC7A11, TF, EPAS1*, and *GCLC*. These are gene sets with FR genes from module groups 2, 3, and 5. Statistically significant results (−log_10_ adjusted *p*-value ≤ 0.05) are marked with stars (*). Abbreviations: pcw, post conceptional weeks; mos, postnatal months; yrs, postnatal years.

Combined with the top 50 correlated genes in the hDLPFC development network based on the calculated adjacency, those 22 FR genes were expanded into 22 FR gene sets composed of 51 genes. Then functional annotation of those gene sets was conducted by overlaying BrainSpan data, a brain developmental transcriptome data set spanning from 8 pcw to 40 postnatal years [87], on the 22 FR gene sets. As illustrated, there was a distinct temporal enrichment pattern of ferroptosis-associated genes during the brain development (Figure 2).

As described in the overview section, neuronal populations go through their most rapid proliferation and organization in prenatal brain development. Though they also begin prenatally, proliferation and organization of glial cells ramp up after birth and the same is true for the last stage of CNS maturation, myelination. Taking this shift of neuron–glia activation into account, it is reasonable to deduce that gene sets of *NCOA4, POR,* and *PROM2* largely modulate processes like neurogenesis and the others regulate the proliferation and migration of both neuron and glial progenitors and also myelination. Above all, *YAP1, NFE2L2, HMOX1, SLC7A11,* and *EPAS1* are of particular note in that they were upregulated at 37 pcw. The literature confirmed that the formation of tertiary sulci, which starts at 36 pcw, is responsible for cognitive development. Thus enriched patterns of five gene sets at 37 pcw imply the engagement of those genes in cognitive development, yet whether they are directly associated with cognition requires further investigation. Additionally, *NCOA4* and *YAP1* were enriched between 8 pcw and 16 pcw, when the most neurons within a brain are produced.

**Figure 3:**
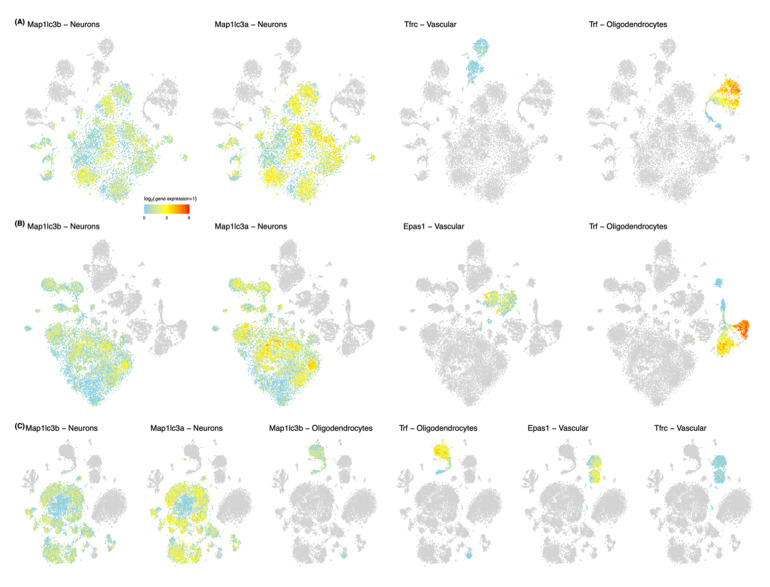
Ferroptosis gene expressions in the cortices of the adolescent mouse brain. Distributions of FR genes were described for various cell types in the mouse brain. Cell types, including neurons, oligodendrocytes, or other non-neuronal cells are described as a cluster of cells using the t-distributed stochastic neighbor embedding (t-SNE), a probabilistic method for visualizing high dimensional single cell RNA-sequencing data. Among FR genes that are converted to mouse genes, five genes (*Epas1*, *Map1lc3a*, *Map1lc3b*, *Tfrc*, *Trf*) with postnatal rising trajectory were used. Only clusters with markers that intersected with FR genes are colored, according to the expression levels, which are log_2_ transformed. Clusters that are not of interest in each plot are colored in light gray. (**A**) t-SNE plot in anterior cortex; (**B**) middle cortex; (**C**) posterior cortex.

**Figure 4:**
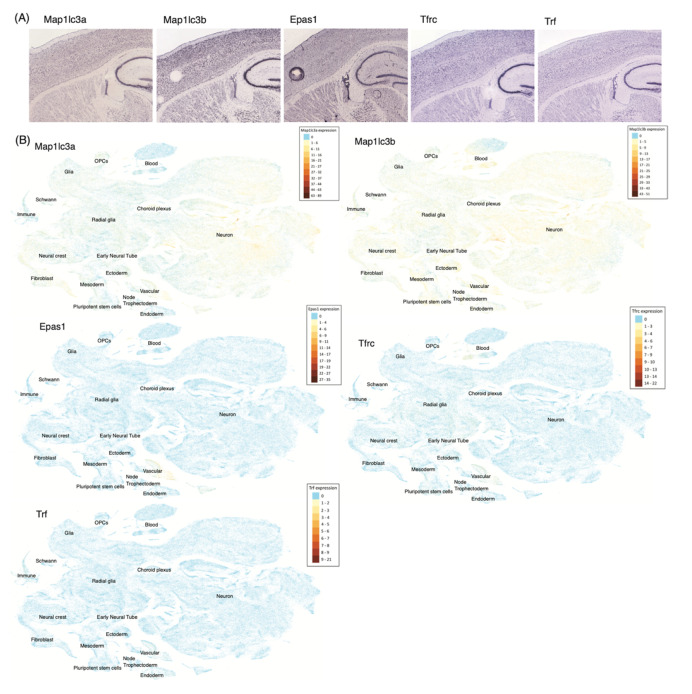
Ferroptosis gene expressions in the mouse brain. (**A**) Results of in situ hybridization (ISH) experiments localizing FR gene expressions in the cortices of the P56 mouse brain of Allen Brain ISH Atlas [137]. (**B**) t-SNE plot of cells expressing FR genes across the developing mouse brain, using the UCSC Cell Browser and the single cell RNA-sequencing data of Manno et al. [138]. Cell types are annotated inside the plots.

To further examine our findings, we visualized the expression of FR genes in the single cell analysis of Zeisel et al. [134] (Figure 3). As displayed, each FR gene showed cell type specificity in adolescent mice. In the anterior cortex, *MAP1LC3B, MAP1LC3A, TFRC,* and *TF* exhibited appreciable expression. *MAP1LC3B* and *MAP1LC3A* were highly expressed in neurons, whereas *TFRC* was in vascular cells and *TF* was in oligodendrocytes. In a similar manner, significant expression was observed for *MAP1LC3B, MAP1LC3A,* and *TF* in the middle cortex except for *EPAS1,* of which expression is largely distributed in vascular cells. Again, in the posterior cortex, the same genes, *MAP1LC3B, MAP1LC3A, EPAS1, TFRC,* and *TF,* showed detectable expression, but this time *MAP1L3B* was also vastly expressed in oligodendrocytes. This intersection of postnatal FR genes and cortex cell type markers can be ascribed to their spatiotemporal functional roles in brain development. This outcome is commensurate with the previous studies [123,135,136]. In situ hybridization (ISH) in Lein et al. [137] and single cell visualization in Manno et al. [138] of those five genes, *MAP1LC3A, MAP1LC3B, EPAS1, TFRC*, and *TF,* support this discovery as well (Figure 4).

## 6. Conclusions

Ferroptosis, a unique form of regulated cell death that is regulated by multiple metabolic pathways in the cell, recently has been in the limelight. Considering the implication of ferroptosis in miscellaneous neurological disorders, to explore its underlying genetic mechanism and prospective role in the brain development is of practical value.

In this review, we assessed the involvement of 42 ferroptosis genes in the brain development. Within the gene co-expression network of hDLPFC development, we identified 22 genes clustered as certain modules. Of 22, 12 genes with non-transitional trajectory were roughly characterized by the maintenance of basic cellular function, like RNA processing, while the other 10 genes with postnatal trajectory were portrayed by their enrichment pattern in neuron and glial cells. Stress being laid on the differential expression level along the course of brain development, we ruled out the non-transitional trajectory group and ran downstream analysis exclusively for 10 genes in the postnatal trajectory group. As a result, upregulated expression of 10 genes at critical points in which neuronal and non-neuronal cells proliferate was observed with *YAP1, NFE2L2, HMOX1, SLC7A11,* and *EPAS1* also upregulated at 37 pcw, a decisive period of cognitive development. Further, gene expression of *MAP1LC3A, MAP1LC3B, EPAS1, TFRC,* and *TF* in mice brains substantiated that those genes are associated with the brain development or at least relate to neuron and glia.

The need for investigating the association between brain development and ferroptosis is more important than ever. To fully comprehend the genetic background of this association, future studies are warranted and scrutinizing the constitutive roles of genes—in particular, the 10 genes we identified—implicated in ferroptosis may help us understand the pathogenesis of many neurological diseases. Also, we expect that integrating single cell data in neurodegenerative disease models such as Niemann–Pick Disease, Type C1 [139] into this analysis will unveil ferroptosis genetic hallmarks under the neurological disease condition.

## Figures and Tables

**Table 1:** List of ferroptosis genes in human dorsolateral prefrontal cortex development.

Gene Symbol	Trajectory Group	Module Assigned	Module Description	Cellular Group
FR-inducing genes				
*NCOA4*	Non- transitional	M5	RNA processing	No cellular specificity
*ATF4*		M6	RNA processing	
*VDAC2*				
*VDAC3*				
*POR*		M7	RNA processing	
*LPCAT3*		M9	Chromosome organization	
*MAP1LC3B*	Postnatal	M16	Ribose phosphate metabolic process	Excitatory neuron
*TFRC*				
*MAP1LC3A*		M17	Macroautophagy	
*HMOX1*		M18	Immune effector process	Non-neuronal cell
*YAP1*				
*EPAS1*		M19	Synaptic signaling	Excitatory neuron, Non-neuronal cell
*TF*				
**FR-suppressing genes**				
*GSS*	Non- transitional	M6	RNA processing	No cellular specificity
*PROM2*		M9	Chromosome organization	
*AIFM2*		M12	Regulation of ion transmembrane transport	
*FTL*		M14	Mitochondrion organization	
*GPX4*				
*SLC3A2*				
*NFE2L2*	Postnatal	M18	Immune effector process	Non-neuronal cell
*SLC7A11*				
*GCLC*		M19	Synaptic signaling	Excitatory neuron, Non-neuronal cell
